# Phase I dose-escalation study of milademetan in patients with relapsed or refractory acute myeloid leukemia

**DOI:** 10.1007/s12185-022-03464-z

**Published:** 2022-10-19

**Authors:** Naohiro Sekiguchi, Senji Kasahara, Toshihiro Miyamoto, Toru Kiguchi, Hitoshi Ohno, Taiga Takagi, Masaya Tachibana, Hiroyuki Sumi, Yasuyuki Kakurai, Tomonari Yamashita, Kensuke Usuki

**Affiliations:** 1 National Hospital Organization Disaster Medical Center, Tokyo, Japan; 2 Gifu Municipal Hospital, Gifu, Japan; 3 Kyushu University Hospital, Fukuoka, Japan; 4 Dokkyo Medical University Saitama Medical Center, Saitama, Japan; 5 Chugoku Central Hospital, Hiroshima, Japan; 6 Tenri Hospital, Nara, Japan; 7 Daiichi Sankyo Co., Ltd, Tokyo, Japan; 8 Department of Hematology, NTT Medical Center Tokyo, 5‑9‑22 Higashi‑Gotanda, Shinagawa‑ku, Tokyo, 141‑8625 Japan

**Keywords:** Acute myeloid leukemia, MDM2 protein, Milademetan, Phase-1 clinical trial, Tumor suppressor protein p53

## Abstract

Long-term survival in patients with acute myeloid leukemia (AML) remains low, and current treatment modalities are inadequate. Milademetan (DS-3032, RAIN-32), a small-molecule specific murine double minute 2 inhibitor, has shown a p53 status-dependent antitumor effect in vitro studies. This is the first phase I study report of milademetan monotherapy in relapsed/refractory (R/R) AML patients evaluating the safety, tolerability, pharmacokinetics, and preliminary tumor response for further clinical development. Fourteen patients received 90 (starting dose, *n* = 4), 120 (*n* = 6), or 160 mg (*n* = 4) of oral milademetan once daily in a 14/28 treatment cycle. The median total treatment duration was 1.5 cycles. Dose-limiting toxicity did not occur, and the maximum tolerated dose was not reached. Thus, the recommended dose was defined as 160 mg. The most common adverse events (AEs) were decreased appetite (64.3%), febrile neutropenia (50%), nausea (42.9%), and anemia (35.7%). No deaths or AEs leading to treatment discontinuation occurred. Five serious treatment-emergent AEs occurred in 4 patients. Plasma concentration increased linearly with milademetan dose. However, trends in the safety and efficacy of oral milademetan in patients with R/R AML warrant further clinical investigation. This study can inform future milademetan studies in hematologic malignancies.

**Supplementary Information:**

The online version contains supplementary material available at 10.1007/s12185-022-03464-z.

## Introduction

Acute myeloid leukemia (AML) is a hematological malignancy characterized by an autonomous growth of myeloid blast cells that cannot differentiate or further develop into mature white blood cells. Moreover, it has a diverse clinical presentation [1] and is the most common type of acute leukemia among adults, comprising ~ 70% of all leukemia cases in Japan [2, 3].

While AML is now treatable and curable, the long-term survival rate after complete remission (CR) with chemotherapy remains low at about 40%, and the relapse rate remains high [4]. Patients with relapsed or refractory (R/R) AML in Japan are currently treated with chemotherapy, gemtuzumab ozogamicin, or FLT3 inhibitors [5], although none of these therapies provide a satisfactory therapeutic effect [1]. Thus, optimizing therapeutic effects is needed for R/R disease in patients with AML.

Intermolecular interactions with murine double minute 2 (MDM2) frequently inhibit the tumor suppressor protein p53 activity in human tumors harboring wild-type p53. MDM2 promotes p53 nuclear export or p53 degradation via the ubiquitin–proteasome pathway, which maintains low p53 activity levels [6]. In addition, ~ 90% of AML patients have wild-type *TP53* in their leukemia cells, and p53 activity is kept low by overexpressed MDM2 binding [7]. MDM2 overexpression disrupts the balance between MDM2 and p53 in humans, reducing p53 function and potentially allowing tumorigenesis and tumor growth [8]. Therefore, inhibiting the interaction between MDM2 and p53 in tumor cells containing wild-type p53 could sustain and increase p53 activity and its resultant antitumor effects [9, 10].

Milademetan (DS-3032, RAIN-32) is an orally administered small-molecule MDM2 inhibitor, and in vitro studies show that it inhibits the MDM2–p53 interaction and has a p53 status-dependent antitumor effect [11, 12]. A phase I dose-escalation study of milademetan in Japanese patients was previously conducted in advanced solid tumors [13]. Thus, the potential clinical impact of milademetan in hematological malignancies, including AML, necessitates additional studies. This multicenter phase I study sought to assess the safety, tolerability, pharmacokinetics (PK), and preliminary tumor response of milademetan monotherapy in R/R AML patients in Japan (NCT03671564, DS3032-A-J104).

## Materials and methods

### Study design and treatment

This phase I, open-label, and dose-escalation study conducted at eight centers in Japan recruited patients with R/R AML. Four fixed-dose milademetan schedules were planned (Supplementary Fig. 1A). Increases in the milademetan dose were guided by the modified continual reassessment method (mCRM), with a planned maximum dose of 160 mg/day (Supplementary Fig. 1B). Each cohort (90, 120, and 160 mg) consisted of three to six patients. The first cycle was defined as the dose-limiting toxicity (DLT) evaluation period where patients required hospitalization. The number of treatment cycles was not specified, and the study treatment for each patient was continued unless the discontinuation criteria were met.

A phase I study conducted in the USA (NCT02319369) administered oral milademetan once daily for 14 days, followed by 14 days of rest in a 28-day cycle (QD 14/28-day schedule, data not published), with other treatment schedules for hematologic malignancies like R/R AML and high-risk myelodysplastic syndrome. The maximum tolerated dose (MTD) was 160 mg, while it was 90 mg on a QD 21/28-day schedule for the Japanese phase I study (Japic CTI-142693) in patients with solid tumors or lymphomas [13]. This dose-escalation study (schedule A) for R/R AML was planned based on US and Japanese studies, with a starting dose of 90 mg QD in a 14/28-day schedule for R/R AML.

In this study, we only assessed the patients with schedule A‒milademetan was administered once daily on days 1–14 followed by a 14-day rest in a 28-day cycle.

### Study population

This study included patients ≥ 20 years old with R/R AML and those with a history of myelodysplastic syndrome. Also eligible were patients who failed to achieve remission with at least one cycle of prior induction therapy, who relapsed after achieving remission with prior therapy, could not achieve persistent remission with standard treatment, had failed to complete potentially curative treatment, or had no treatment options with expected therapeutic efficacy. The Eastern Cooperative Oncology Group performance status (ECOG PS) scores for these patients ranged from 0 to 2. This study excluded those with a diagnosis of acute promyelocytic leukemia, chronic myeloid leukemia in a blast crisis (positive for a breakpoint cluster region-c-Abelson [BCR-ABL] fusion gene), history of or concurrent central nervous system leukemia, history of hematopoietic stem cell transplant within 60 days before the initiation of this study, graft-versus-host disease, and grade ≥ 2 clinically significant or irreversible nonhematological toxicity related to transplantation.

Although confirmation of *TP53* wild-type status was not required before initiating milademetan treatment, the investigators and patients were notified of the availability of the genotyping results. Patients were eligible to continue the study treatment if the principal investigator’s clinical judgment indicated benefit from treatment once test results after milademetan administration revealed that a patient’s malignancy contained a *TP53* gene mutation.

Patients were disqualified from the study if an apparent disease progression was noted, adverse events (AEs) made it difficult to continue milademetan treatment, consent was withdrawn, milademetan was administered on < 75% of the specified dosing days, or the investigators deemed it inappropriate to continue the study. In the case of the latter, the investigators always recorded the date and reasons for discontinuation.

### Objectives

This study primarily aimed to evaluate the safety, tolerability, and MTD of milademetan monotherapy in Japanese patients with R/R AML. The secondary objective was to determine the PK profile and tumor response. Additionally, the recommended dose of the further study was determined. Furthermore, the *TP53* genomic status was investigated exploratorily as a potential resistance mechanism. The pharmacodynamic effects of milademetan on macrophage inhibitory cytokine-1 (MIC-1) serum levels were also evaluated.

### Safety evaluation

Relative dose intensity (in percentage) was calculated as a proportion of dose intensity/planned dose intensity (proportion of dose administered/total treatment duration). Safety assessments were completed by documenting the AEs, ECOG PS, laboratory data, body weight, vital signs, and the results of 12 lead ECGs. Serious adverse events (SAEs) were defined as any AEs that caused mortality, were life-threatening, required hospitalization or prolonged existing hospitalization, caused disability or incapacity, or represented other medical conditions. Patients were followed up until the AEs resolved.

Patients were evaluated for DLT during the first 28 days of cycle 1, defined as any nonhematological AEs of grade ≥ 3 unless attributed to the primary disease course, complications, or concomitant medications. Nonhematological toxicities classified as DLTs were grade 4 AST/ALT, grade 3 AST/ALT lasting ≥ 3 days, grade 3 AST/ALT with grade ≥ 2 total bilirubin, or an inability to complete at least 75% of the prescribed milademetan doses in cycle 1 (28 days) due to grade ≥ 2 events. Hematological toxicities classified as DLTs included failure to recover neutrophil and platelet counts to ≥ 500/mm^3^ and ≥ 20,000/mm^3^, respectively, that delayed the initiation of cycle 2 for > 2 weeks. DLT evaluation findings were used to determine the MTD. AEs that were not classified as DLTs included grade 3 fatigue of < 3 days; grade 3 nausea or vomiting that resolved to grade ≤ 2 within 2 days after antiemetic therapy; grade 3 diarrheas that resolved to grade ≤ 2 within 2 days after antidiarrheal therapy; and alopecia, febrile neutropenia, and transient laboratory abnormalities either without symptoms or requiring continuous treatment. Examples of non-DLT AEs were grade ≥ 3 alkaline phosphatases, uric acid, amylase, lipase, or hyponatremia that resolved within 72 h of onset. The Common Terminology Criteria for Adverse Events (version 5.0) were used for grading, and each AE was coded using the Medical Dictionary for Regulatory Activities (version 21.1). Criteria for drug dose reduction or interruption are described in Supplementary material.

### Pharmacokinetic assessment

Blood samples were collected for PK assessment before the first dose and at 1, 2, 3, 6, and 8 h after oral milademetan on days 1 and 14 of cycle 1, before the dose on days 2 and 8 of cycle 1, and before the dose and at 3 h after milademetan on day 1 of cycle 2. Milademetan concentrations were determined using a validated HPLC/MS method. The lower limit of quantification was 0.500 ng/mL. Noncompartmental analysis was used to calculate the PK parameters from milademetan plasma concentrations. In addition, the Phoenix WinNonlin 8.1 software (Certara, Princeton, NJ, USA) was used for PK analysis.

### Pharmacodynamic assessment

To investigate biomarkers that could be potentially linked to the milademetan mechanism of action, blood, bone marrow, and serum were collected. *TP53* tumor genotyping was done using bone marrow and blood samples collected before milademetan administration and at the end of treatment. *TP53* genomic status was determined using a targeted next-generation sequencing following the reference human genome sequence (hg19). QIAamp^®^ DSP FFPE Kit (Qiagen, Hilden, Germany) was used to extract DNA. Quantified DNA samples were processed by polymerase chain reaction (PCR) amplification using custom primer sets of all 11 *TP53* exons. Libraries were prepared from purified PCR pools using Illumina TruSeq kits^™^ (Illumina, San Diego, CA, USA) and analyzed using the MiSeq platform^™^ (Illumina). Raw reads were aligned against hg19, and data sets were analyzed using a program developed in-house for genomic aberrations (i.e., mutations, deletions, and insertions) detection. Quantikine^®^ ELISA Human GDF-15 immunoassay (R&D Systems, Inc., Minneapolis, MN, USA), employing the quantitative sandwich enzyme immunoassay technique, was used to determine serum MIC-1, also known as growth and differentiation factor-15 (GDF-15). Serum MIC-1 samples were collected before the dose on days 1, 2, 8, 14, and 22 of cycle 1 and before the dose on day 1 of cycle 2.

### Efficacy assessment

Bone marrow and peripheral blood results were used to assess the antitumor effect of milademetan. AML response criteria were defined as shown in Supplementary Table 1. A morphologic leukemia-free state (MLFS) was defined as bone marrow blasts < 5% in the absence of blasts with Auer rods and the absence of extramedullary leukemia. The presence/absence of hematological recovery or blood transfusion was not considered. Stable disease (SD) was defined as an absence of CR, CR with incomplete hematologic recovery (CRi) or partial hematologic recovery (CRh), partial remission (PR), or MLFS, provided that the criteria for the progressive disease were not met. SD had to last at least 3 months (assessed as SD more than thrice in a row) after initiating milademetan treatment. SD < 3 months was deemed not applicable. The best-measured response was defined as the best overall response (all time points after the start of study treatment until the end of treatment). The composite CR (CRc) duration was defined as the time between when the criteria for CRc (CR + CRi + CRh) were first met and when a relapse was first confirmed.

### Definitions and statistical analysis

All patients who received at least one milademetan dose were included in the safety analysis set. The MTD analysis set included patients who received at least one milademetan dose, had no missing data for examination or observation during the DLT evaluation period, and had a completed DLT evaluation. Without DLTs, the included patients received ≥ 75% milademetan of the specified days in cycle 1. Patients who experienced DLT during the DLT evaluation period and resumed the study treatment at a lower dose were included in the initial dose cohort. The efficacy analysis set included all patients who received at least one milademetan dose and underwent efficacy evaluation at least once after treatment initiation. The PK analysis set comprised patients who received at least one milademetan dose, for whom a PK sample was collected at least once after initiating treatment, and who had available PK measurement data. The biomarker analysis set included all patients who received at least one milademetan dose, whose specimens were collected for biomarker studies, and who had available measurement data. Following the first cycle, doses for subsequent cohorts were guided by an mCRM based on a Bayesian logistic regression model incorporating the escalation with the overdose control principle, clinical evaluation of the toxicological profile, and pharmacokinetics and pharmacodynamics information. Continuous variables were summarized with mean, standard deviation, and minimum, median, and maximum values, while categorical variables were summarized with a frequency table.

In addition to descriptive statistics, the geometric mean and geometric coefficient of variation were used to describe the PK parameters. All analyses were conducted using SAS^®^ software (version 9.4; SAS Institute Inc., Cary, NC, USA). The time courses of mean plasma milademetan concentrations on days 1 and 14 of cycle 1 (linear scale) in the QD 14/28-day schedule were used for pharmacokinetic analysis. The best response was measured as CR, CRh, CRi, or PR. For efficacy analysis, a waterfall plot of the best percent change from baseline blast count in bone marrow aspirate on the QD 14/28-day schedule was drawn to assess the blast count reduction. In addition, line plots of individual serum MIC-1 levels were drawn in the QD 14/28-day schedule for the pharmacodynamics analysis set.

## Results

This study protocol primarily evaluated the QD 14/28 milademetan schedule in AML patients. The QD 14/28 treatment schedule was required in this study to participate in another phase I study in combination with quizartinib (NCT03552029) [14], and this schedule demonstrated safety and tolerability. Thus, only schedule A was evaluated in this study.

### Patients’ disposition and baseline characteristics

**Fig. 1:**
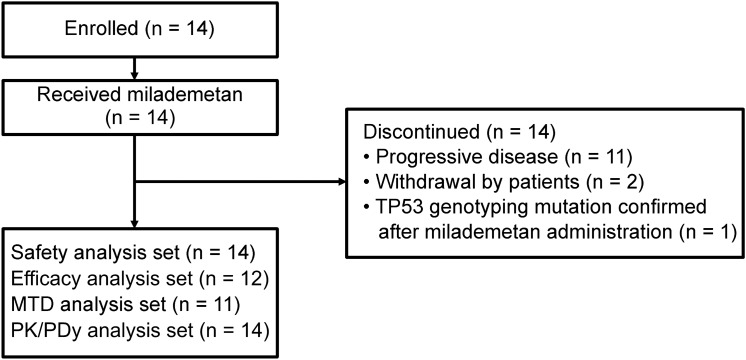
Patient disposition *MTD* Maximum tolerated dose, *PDy* pharmacodynamics, *PK* pharmacokinetics

Following written informed consent, 14 patients were enrolled in this study, of which four, six, and four were placed in the 90-, 120-, and 160 mg treatment cohorts, respectively (Fig. 1). The patients’ median age (range) was 72 (42–76) years old, and 11 patients were ≥ 65 years old. All patients had an ECOG PS of 0 or 1. Table 1 shows the baseline characteristics of patients who received milademetan in daily doses of 90, 120, and 160 mg. Three patients in the 120-mg cohort were excluded from the DLT assessment because the DLT assessment could not be completed due to disease progression before the assessment period ended. Thus, 11 of the 14 patients in the safety analysis set were included in the MTD analysis set.

**Table 1:** Baseline characteristics of the study population

Parameter statistic/category	90 mg/day (*n* = 4)	120 mg/day (*n* = 6)	160 mg/day (*n* = 4)	Total (*n* = 14)
Median age, years (range)	74.5 (57–76)	70.0 (42–75)	71.5 (65–75)	72.0 (42–76)
Male, n (%)	4 (100.0)	5 (83.3)	1 (25.0)	10 (71.4)
Median height, cm (range)	169.5 (162–176)	161.5 (153–173)	157.0 (153–164)	162.5 (153–176)
Median body weight, kg (range)	70.30 (59.8–84.7)	46.05 (41.7–68.6)	51.85 (42.8–72.2)	56.20 (41.7–84.7)
ECOG PS, *n* (%)				
0	2 (50.0)	3 (50.0)	2 (50.0)	7 (50.0)
1	2 (50.0)	3 (50.0)	2 (50.0)	7 (50.0)
Chromosomal abnormalities, *n* (%)				
RUNX1-RUNX1T1	0 (0.0)	2 (33.3)	2 (50.0)	4 (28.6)
Not applicable	4 (100)	4 (66.7)	2 (50.0)	10 (71.4)
Acute myeloid leukemia, NOS, *n* (%)				
AML with maturation	1 (25.0)	2 (33.3)	2 (50.0)	5 (35.7)
Acute myelomonocytic leukemia	1 (25.0)	1 (16.7)	0 (0.0)	2 (14.3)
Acute monoblastic and monocytic leukemia	0 (0.0)	0 (0.0)	1 (25.0)	1 (7.1)
Not applicable	2 (50.0)	3 (50.0)	1 (25.0)	6 (42.9)
Prior transplants, *n* (%)	0 (0.0)	2 (33.3)	0 (0.0)	2 (14.3)

*AML* acute myeloid leukemia, *ECOG* *PS* eastern cooperative oncology group performance status, *WHO* world health organization

Not applicable indicates the patients that WHO classification could not be applicable based on Investigator discretion

### Safety

The median (minimum and maximum) total treatment duration of the 14 patients was 1.5 (1 and 8) cycles. The median (minimum and maximum) relative dose intensity was 100% (92.9% and 100%), 100% (7.1% and 100%), and 92.9% (70.7% and 100%) in the 90-, 120-, and 160 mg cohorts, respectively. A summary of TEAEs is provided in Table 2. All grades of treatment-emergent AEs (TEAEs) that occurred in ≥ 20% of the 14 total patients or any grade ≥ 3 TEAEs are listed in Table 3. The most common TEAEs were decreased appetite, febrile neutropenia, nausea, and anemia in nine (64.3%), seven (50%), six (42.9%), and five (35.7%) patients, respectively, regardless of causality. Moreover, grade ≥ 3 TEAEs were reported in 10 (71.4%) of 14 patients. Grade 4 TEAEs included decreased platelet count (three patients), neutropenia and thrombocytopenia (two patients each), and decreased neutrophil count (one patient).

**Table 2:** Summary of treatment-emergent adverse events (TEAEs)

*n* (%)	90 mg/day (*n* = 4)	120 mg/day (*n* = 6)	160 mg/day (*n* = 4)	Total (*n* = 14)
TEAE	4 (100.0)	5 (83.3)	4 (100.0)	13 (92.9)
Serious TEAE	2 (50.0)	2 (33.3)	0 (0.0)	4 (28.6)
TEAE by CTCAE grade				
≤ 2	0 (0.0)	3 (50.0)	0 (0.0)	3 (21.4)
≥ 3	4 (100.0)	2 (33.3)	4 (100.0)	10 (71.4)

Treatment-emergent adverse event (TEAE) is an adverse event that occurs after the first administration or worsens relative to the pretreatment state

Coded with Medical Dictionary for Regulatory Activities (MedDRA) Version 21.1

**Table 3:** Most frequent treatment-emergent adverse events (TEAEs) [≥ 20% (all grades) or any (grade ≥ 3)]

TEAE, *n* (%)	90 mg/day (*n* = 4)		120 mg/day (*n* = 6)		160 mg/day (*n* = 4)		Total (*n* = 14)	
	Grade ≥ 3	All grades	Grade ≥ 3	All grades	Grade ≥ 3	All grades	Grade ≥ 3	All grades
Decreased appetite	0 (0.0)	3 (75.0)	0 (0.0)	3 (50.0)	1 (25.0)	3 (75.0)	1 (7.1)	9 (64.3)
Febrile neutropenia	3 (75.0)	3 (75.0)	1 (16.7)	1 (16.7)	3 (75.0)	3 (75.0)	7 (50.0)	7 (50.0)
Nausea	0 (0.0)	1 (25.0)	0 (0.0)	2 (33.0)	0 (0.0)	3 (75.0)	0 (0.0)	6 (42.9)
Anemia	2 (50.0)	2 (50.0)	1 (16.7)	1 (16.7)	2 (50.0)	2 (50.0)	5 (35.7)	5 (35.7)
Thrombocytopenia	2 (50.0)	2 (50.0)	1 (16.7)	1 (16.7)	1 (25.0)	1 (25.0)	4 (28.6)	4 (28.6)
Pneumonia	2 (50.0)	2 (50.0)	0 (0.0)	1 (16.7)	1 (25.0)	1 (25.0)	3 (21.4)	4 (28.6)
Hypokalemia	0 (0.0)	0 (0.0)	0 (0.0)	2 (33.3)	1 (25.0)	2 (50.0)	1 (7.1)	4 (28.6)
Vomiting	0 (0.0)	0 (0.0)	0 (0.0)	1 (16.7)	0 (0.0)	3 (75.0)	0 (0.0)	4 (28.6)
Platelet count decreased	1 (25.0)	1 (25.0)	1 (16.7)	1 (16.7)	1 (25.0)	1 (25.0)	3 (21.4)	3 (21.4)
Diarrhea	0 (0.0)	0 (0.0)	0 (0.0)	1 (16.7)	1 (25.0)	2 (50.0)	1 (7.1)	3 (21.4)
Malaise	0 (0.0)	0 (0.0)	0 (0.0)	2 (33.3)	0 (0.0)	1 (25.0)	0 (0.0)	3 (21.4)
Dry mouth	0 (0.0)	1 (25.0)	0 (0.0)	0 (0.0)	0 (0.0)	2 (50.0)	0 (0.0)	3 (21.4)
Stomatitis	0 (0.0)	1 (25.0)	0 (0.0)	1 (16.7)	0 (0.0)	1 (25.0)	0 (0.0)	3 (21.4)
Neutropenia	2 (50.0)	2 (50.0)	0 (0.0)	0 (0.0)	0 (0.0)	0 (0.0)	2 (14.3)	2 (14.3)
Leukopenia	1 (25.0)	1 (25.0)	0 (0.0)	0 (0.0)	0 (0.0)	0 (0.0)	1 (7.1)	1 (7.1)
Neutrophil count decreased	1 (25.0)	1 (25.0)	0 (0.0)	0 (0.0)	0 (0.0)	0 (0.0)	1 (7.1)	1 (7.1)
White blood cell count decreased	1 (25.0)	1 (25.0)	0 (0.0)	0 (0.0)	0 (0.0)	0 (0.0)	1 (7.1)	1 (7.1)
Pharyngitis	0 (0.0)	0 (0.0)	0 (0.0)	0 (0.0)	1 (25.0)	1 (25.0)	1 (7.1)	1 (7.1)
Skin infection	0 (0.0)	0 (0.0)	1 (16.7)	1 (16.7)	0 (0.0)	0 (0.0)	1 (7.1)	1 (7.1)

Treatment-emergent adverse event (TEAE) is an adverse event that occurs after the first administration or worsens relative to the pretreatment state

Coded with Medical Dictionary for Regulatory Activities (MedDRA) Version 21.1

DLT assessment was completed by all 11 patients in the MTD analysis set, and no DLTs were reported in any of the three cohorts during the evaluation period (cycle 1). The MTD was not reached, thus, recommended dose was defined as 160 mg in the 14/28 treatment cycle. The five reported SAEs in four patients included febrile neutropenia (two events) and pneumonia (three events). Both febrile neutropenia events and one pneumonia event were drug-related. No death or AEs that caused treatment discontinuation were noted. All patients discontinued study treatment due to progressive disease (*n* = 11), patient withdrawal (*n* = 2), and *TP53* genotype mutation confirmed after milademetan administration (*n* = 1). One (7.1%) of 14 patients had a TEAE that required dose reduction (acute kidney injury). Three (21.4%) of 14 patients had TEAEs leading to study treatment interruption, including febrile neutropenia (*n* = 2), decreased appetite (*n* = 1), dysgeusia (*n* = 1), diarrhea (*n* = 1), decreased neutrophil count (*n* = 1), and decreased platelet count (*n* = 1). The mean platelet count decreased from the baseline in all cohorts. No clinically relevant changes from baseline or consistent trends were observed in other laboratory parameters (hematology, blood chemistry, or urinalysis), vital signs, or ECG parameters. Summary of neutrophil and platelet counts across all visits were presented in supplementary Table 2.

### Pharmacokinetics

**Fig. 2:**
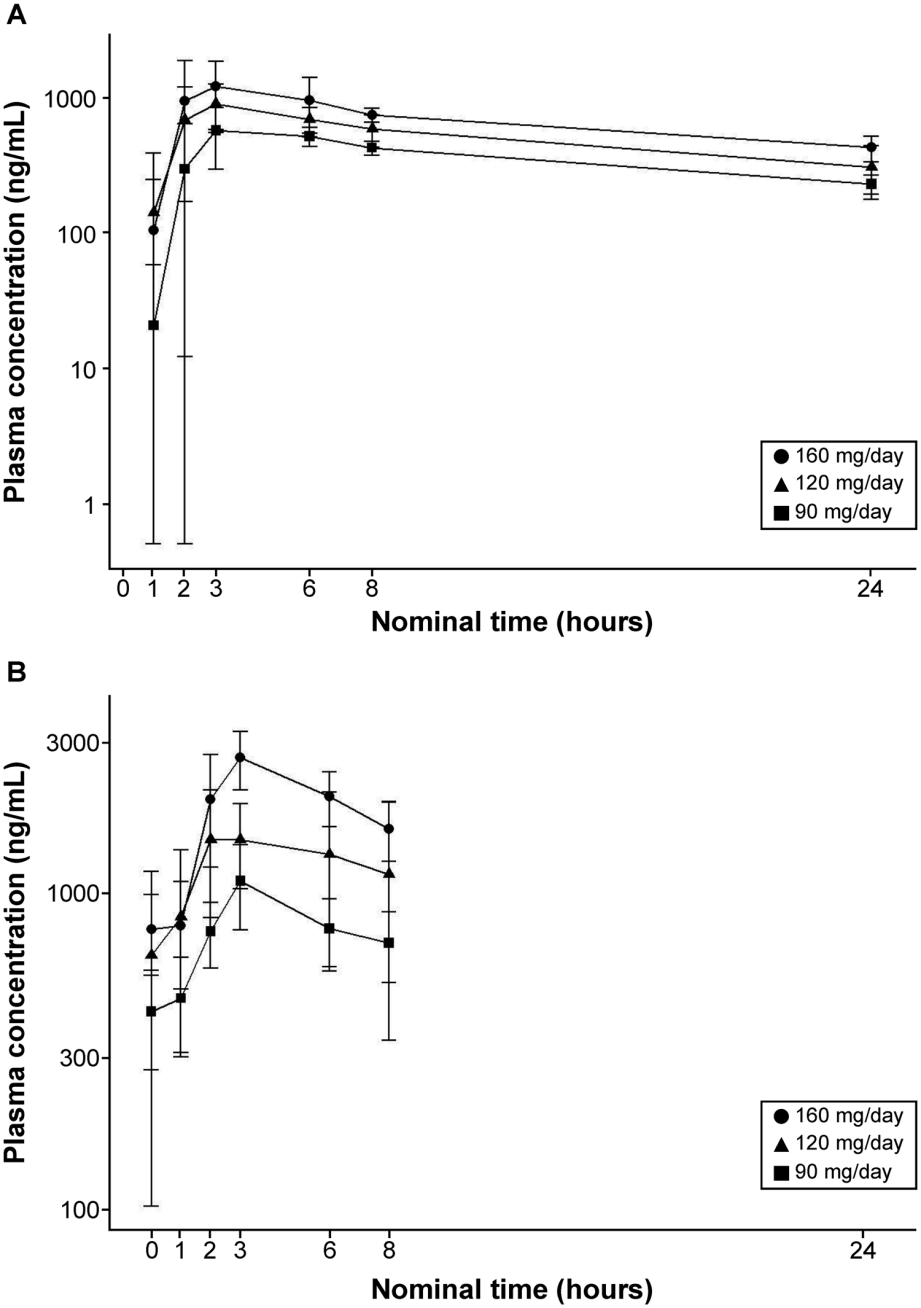
Mean plasma concentration–time curves of milademetan **A** Day 1 of cycle 1; **B** day 14 of cycle 1. Cohort 1, 90 mg 14/28 once daily; cohort 2, 120 mg 14/28 once daily; and cohort 3, 160 mg 14/28 once daily. Data represent the arithmetic mean ± standard deviation

The plasma concentration–time profile of milademetan for days 1 and 14 of cycle 1 in the QD 14/28-day schedule is shown in Fig. 2. Milademetan was rapidly absorbed with a median *T*_max_ of ~ 3–4 h (Table 4), and its mean half-life was ~ 15 h. The increase in *C*_max_ and area under curve (AUC) values were linear and dose-proportional across the dose range tested after both single and multiple dosing, indicating the linear PK of milademetan in patients with R/R AML (Supplementary Figs. 1 and 2). The mean accumulation ratios based on AUC_24h_ were 1.40–1.90 at the dose range tested on Day 14.

**Table 4:** Summary of pharmacokinetic parameters

	90 mg/day	120 mg/day	160 mg/day
Day 1 of cycle 1			
* n*	4^a^	6^b^	4^a^
* C*_max_ (ng/mL)	653 (196)	1,010 (444)	1,380 (540)
* T*_max_ (h)	4.45 (2.97, 5.88)	3.13 (1.92, 5.92)	2.54 (2.00, 8.00)
AUC_8h_ (ng h/mL)	3,170 (905)	4,940 (1,590)	6,560 (3,200)
AUC_24h_ (ng h/mL)	8,640 (2,210)	12,100 (4,080)	15,000 (1,380)
AUC_last_ (ng h/mL)	8,060 (1,400)	11,300 (3,450)	14,900 (4,400)
AUC_inf_ (ng h/mL)	13,700 (4,100)	18,600 (8,600)	22,700 (5,080)
* t*_1/2_ (h)	15.9 (1.35)	14.1 (4.27)	14.6 (4.36)
Day 14 of cycle 1			
* n*	4	5	4
* C*_max_ (ng/mL)	1,090 (327)	1,680 (657)	2,710 (540)
* C*_trough_ (ng/mL)	422 (147)	635 (533)	769 (221)
* T*_max_ (h)	3.02 (2.97, 3.08)	2.98 (2.08, 5.92)	3.03 (1.87, 3.17)
AUC_8h_ (ng h/mL)	6,220 (1,480)	10,000 (4 750)	15,300 (3 320)

*AUC*_*8h*_ area under the plasma concentration–time curve during 8 h, *AUC*_*24h*_ area under the plasma concentration–time curve during 24 h, *AUC*_*last*_ area under the plasma concentration–time curve up to the last quantifiable time, *AUC*_*inf*_ area under the plasma concentration–time curve up to infinity, *C*_*max*_ maximum plasma concentration, *C*_*trough*_ trough plasma concentration, *T*_*max*_ time to reach maximum plasma concentration, *t*_*1/2*_ (h) termination elimination half-life in hours

The means (standard deviations) are provided for the PK parameters except for *T*_max_, for which the median (minimum, maximum) values are presented

^a^Values of AUC_24h_, AUC_inf_, and *t*_1/2_ for two patients were excluded from the summary

^b^Values of AUC_24h_, AUC_inf_, and *t*_1/2_ for one patient were excluded from the summary

### Pharmacodynamics (biomarker)

#### TP53 genomic mutation status

A mutation in the *TP53* gene was found in the blood samples of two (one in each of the 90- and 160-mg cohorts) of 14 patients and in the bone marrow aspirates of three (one each in all three cohorts) of 14 patients before milademetan administration. The *TP53* gene mutation was present in the blood samples of three (two and one in the 120- and 160-mg cohorts, respectively) of 12 patients and bone marrow aspirates for four (two in the 120-mg cohort and one each in the 90- and 160-mg cohorts) of eight patients at the end of treatment (Supplementary Table 3). The best percent change of blast count from baseline to the end of treatment by *TP53* status at the baseline in each patient’s bone marrow is shown as a waterfall plot (Supplementary Fig. 3). *TP53* variant allele frequency were about 10–50%.

#### MIC-1 status

**Fig. 3:**
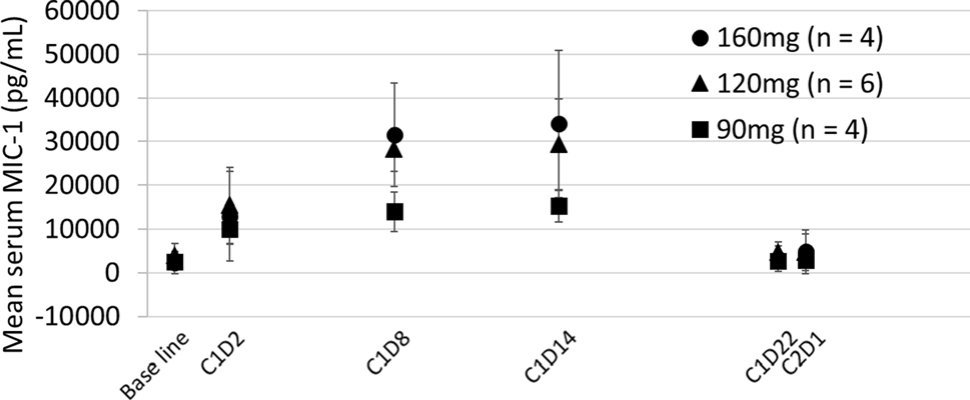
Mean macrophage inhibitory cytokine-1 (MIC-1) serum level *C* cycle, *D* day, *MIC*-1 macrophage inhibitory cytokine-1

MIC-1 is a secreted p53 downstream gene product used as a pharmacodynamic biomarker for p53 activation [15]. The mean serum MIC-1 levels increased from baseline in all cohorts after milademetan administration. The serum MIC-1 level, in most patients, reached a peak on day 8 or 14 of cycle 1 and then decreased to near baseline levels on day 22 of cycle 1 or later (Fig. 3). The peak MIC-1 levels in the 120- and 160-mg cohorts tended to be higher than those in the 90-mg cohort.

### Efficacy

None of the patients had CR, CRh, CRi, or PR. MLFS and SD were observed in two (16.7%) and one (8.3%) of 12 patients, respectively (Table 5), and their *TP53* status at baseline were wild. In 12 patients, the mean (standard deviation) best percentage change from baseline in blast count was − 6.5% (59.0). Four (33.3%) patients had a ≥ 50% reduction in baseline blast count in the bone marrow aspirate (Supplementary Fig. 3).

**Table 5:** Efficacy results with milademetan administration

Best response^a^ (n, %)	90 mg/day (*n* = 4)	120 mg/day (*n* = 5)	160 mg/day (*n* = 3)	Total (*n* = 12)
Morphologic leukemia-free state	0 (0.0)	1 (20.0)	1 (33.3)	2 (16.7)
Stable disease	1 (25.0)	0 (0.0)	0 (0.0)	1 (8.3)
Progressive disease	1 (25.0)	1 (20.0)	0 (0.0)	2 (16.7)
Not applicable	2 (50.0)	3 (60.0)	2 (66.7)	7 (58.3)

^a^Complete remission (CR), CR with incomplete hematologic recovery (CRi), and CR with partial hematologic recovery were not observed (CRh)

## Discussion

This is the first report to evaluate the safety and tolerability of milademetan monotherapy administered in multiple doses for R/R AML patients. Of the 14 enrolled patients, 14, 12, and 11 were included in the safety, efficacy, and MTD analysis sets, respectively. The most common TEAEs were decreased appetite (64.3%) and nausea (42.9%). No dose-dependent increase was observed in the frequency of TEAEs that were grade ≥ 3, serious, or led to dose reduction/treatment interruption. No DLTs were noted during the DLT evaluation period (cycle 1), and the MTD could not be determined, thus, recommended dose was defined as 160 mg in this study (QD 14/28-day schedule).

The safety results of this study demonstrated that milademetan was well tolerated up to 160 mg in the QD 14/28-day schedule without any new safety concerns. Gastrointestinal symptoms (e.g., decreased appetite, nausea, and vomiting) are common TEAEs with milademetan as well as other MDM2 inhibitors. In addition, the drug’s safety profile observed in this study is comparable with that of other MDM2 inhibitors [16].

The *C*_max_ and AUC of milademetan increased approximately proportional to dose after single and multiple doses, indicating a linear PK of milademetan over the dose range tested. Serum MIC-1 induction may be the tendency of dose-dependent p53 pathway reactivation by milademetan. In this study, we did not examine the relationship between TEAE and milademetan exposure measures. However, TEAEs were similar across dose levels in this study.

None of the 12 patients in the efficacy analysis set had CR, CRh, CRi, or PR at 90, 120, or 160 mg doses of milademetan. MLFS and SD were observed in two (16.7%) and one (8.3%) patient, respectively, and their *TP53* status at baseline were wild. Four (33.3%) patients had a ≥ 50% reduction of blast count from baseline in the bone marrow aspirate (Supplementary Fig. 3). The correlation between milademetan efficacy and *TP53* status or MIC-1 was not clear from our data because there were no subjects who achieved CR, CRh, CRi, or PR and the sample size was small. More assessments will be needed in future clinical research.

Other phase I studies of milademetan included combination therapies with azacytidine and quizartinib for R/R AML (NCT02319369) [17] and FLT3-ITD AML (NCT03552029) [14], respectively. Thus, optimizing the recommended milademetan dose and the treatment schedule is necessary in addition to developing more effective treatment regimens, including the concurrent use of other treatments.

Previous reports in AML patients revealed that p53 pathway dysfunction is common regardless of p53 mutation status. Therefore, MDM2 could be a therapeutic target [18]. Reis et al. showed the clinical response to idasanutlin (RG7388) in AML patients and associated pretreatment MDM2 protein expression in leukemic blasts. A secondary finding of their study was that MDM2 protein expression from blasts, when used as a biomarker, could help identify R/R AML patients who are likely to benefit from MDM2 inhibitor-based therapy [19]. Furthermore, a multicenter, randomized, double-blind phase III trial using cytarabine with or without idasantulin, another MDM2 inhibitor, in R/R AML is ongoing [20]. Geoffrey and Sarit, in a phase I monotherapy study in the USA and Canada, demonstrated the favorable safety and antileukemic efficacy of an intravenous administration of MDM2 antagonist RO6839921 in patients with AML [16].

This study had some limitations. This is a small-scale (only three to six patients in each cohort), open-label phase I study with an ethnically homogeneous population, hence it is not possible to generalize the results to more large and heterogenous R/R AML groups. Insufficient follow-up period to assess long-term safety, response duration, or survival are other limitations. In addition, MDM2 status was not defined as an eligibility criterion in this study because no robust evidence was noted to indicate whether MDM2 status is a suitable predictive biomarker when this study began. Therefore, future clinical trials are needed to comprehensively investigate the efficacy and safety of milademetan.

In conclusion, milademetan was generally safe and well tolerated up to 160 mg in the QD 14/28-day schedule in Japanese patients with R/R AML. MTD was not reached at up to 160 mg with the QD 14/28 schedule in this study. The PK parameters, including *C*_max_ and AUC, indicated a dose-dependent increase in milademetan exposure. Milademetan monotherapy was well tolerated in patients with R/R AML because no DLTs were observed in this study. Additional research is needed to confirm the safety, tolerability, and clinical activity of milademetan in patients with R/R AML with monotherapy or combination chemotherapy dose regimens.

## Supplementary Information

Below is the link to the electronic supplementary material.

Supplementary file1 (DOCX 338 KB)

## Data Availability

Deidentified individual patient data and applicable supporting clinical trial documents may be available upon request at Vivli-Center for Global Clinical Research Data. Daiichi Sankyo will continue to protect the privacy of the clinical trial patients in cases where clinical trial data and supporting documents are provided according to company policies and procedures. Details on data sharing criteria and the procedure for requesting access can be found online (https://vivli.org/ourmember/daiichi-sankyo/).
