# Supplementary material for: Phase I dose-escalation study of milademetan in patients with relapsed or refractory acute myeloid leukemia
Source: Int J Hematol. 2022 Oct 19;117(1):68–77. doi: 10.1007/s12185-022-03464-z (PMC9813109; doi:10.1007/s12185-022-03464-z)
Supplement: Supplementary file 1 — Supplementary file1 (DOCX 338 KB) [file 12185_2022_3464_MOESM1_ESM.docx]

Contents

[**Materials and Methods** 2](#_Toc112939315)

[**Criteria for drug dose reduction or interruption** 2](#_Toc112939316)

[**Supplementary Table 1. Response criteria for acute myeloid leukemia** 3](#_Toc112939317)

[**Supplementary Table 2. Summary of Neutrophils and Platelet Counts in the Safety Analysis Set** 5](#_Toc112939318)

[**Supplementary Table 3. *TP53* status** 6](#_Toc112939319)

[**Supplementary Figure 1. Treatment schedule and study design** 7](#_Toc112939320)

[**A. Doses and dosing schedules** 7](#_Toc112939321)

[**B. Study Design** 8](#_Toc112939322)

[**Supplementary Figure 2. Scatter plots of milademetan dose and AUC for the individual patients** 9](#_Toc112939323)

[**Supplementary Figure 3. Waterfall plot of best percent change from baseline blast count in bone marrow aspirate** 10](#_Toc112939324)

# **Materials and Methods**

## **Criteria for drug dose reduction or interruption**

In the event of a treatment-related toxicity that meets the DLT definition, the investigator or sub-investigator may interrupt, postpone, reduce dose, or discontinue the study treatment. If any of the following toxicities occurred that were not previously described, the investigator or sub-investigator continued the study treatment for as long as the toxicity could be controlled through appropriate intervention: 1) Grade ≥2 non-hematological toxicity (excluding alopecia) unrelated to the primary disease, Grade ≥3 fatigue lasting >48 hours; 2) Grade ≥2 laboratory abnormalities not meeting the definition of DLTs; 3) Hypoplastic marrow (neutrophil count <500 /mm^3^ and platelet count <20,000 /mm^3^).

When it was deemed necessary to interrupt or postpone the study treatment, the interruption or postponement was considered until the toxicity resolved to the following conditions: 1) Neutrophil count ≥500 /mm^3^); 2) Platelet count ≥20,000/mm^3^; 3) Grade ≥2 non-hematological toxicities (excluding alopecia) that improved to Grade ≤1 or resolved to baseline levels.

If the toxicity was confirmed to have improved or resolved within 4 weeks, the study treatment could be resumed. When the study treatment was resumed, a one-level dose reduction from the dose received by the relevant subject prior to experiencing toxicity was considered.

# **Supplementary Table 1. Response criteria for acute myeloid leukemia**

**Best response, CRc rate, and overall response rate (efficacy analysis set)**

| Complete remission: CR^a^ | Bone marrow blasts (<5%), neutrophil count (≥1,000/mm^3^), and platelet count (≥100,000/mm^3^) in the absence of red blood cell (RBC) or platelet transfusion, Auer rods, extramedullary leukemia, and peripheral blasts. |
| --- | --- |
| CR with incomplete hematologic recovery: CRi | Meeting all CR criteria except for neutrophil (<1,000/mm^3^) and platelet (≥100,000/mm^3^) count or neutrophil (≥1,000/mm^3^) and platelet (<100,000/mm^3^) count. |
| CR with partial hematologic recovery: CRh^b^ | Meeting all CR criteria except for neutrophil (>500/mm^3^) and platelet count (>50,000/mm^3^). |
| Partial remission: PR | Neutrophil (≥1,000/mm^3^) and platelet (≥100,000/mm^3^) count in the absence of RBC or platelet transfusion (4 weeks and 1 week, respectively) and decrease in bone marrow and total bone marrow blast percentage by at least 50% and 5%–25%, respectively. |
| Morphologic leukemia-free state: MLFS | Bone marrow blasts (<5%) in the absence of blasts with Auer rods and absence of extramedullary leukemia. The presence/absence of hematologic recovery or blood transfusion is not considered. |
| Stable disease: SD | Absence of CR, CRi, CRh, PR, or MLFS, and the criteria for PD are not met. |
| Progressive disease: PD | Bone marrow blasts increasing >1.5-fold over baseline (e.g., a blast count increase of 35%–>52.5%). At least 15%-point increase in bone marrow blasts in cases with <30% blasts at baseline (e.g., a blast count increase of 20%–35%). Persistent bone marrow blast percentage of >70% over at least 3 months. Cases with an improvement of ≥500/mm^3^ in neutrophil count and/or an improvement of ≥50,000/mm^3^ in platelet count (without transfusion) are excluded.  >1.5-fold increase in peripheral blasts (white blood cell [WBC] × %blasts) to >25,000/mm^3^ in the absence of differentiation syndrome.  New extramedullary leukemia. |
| Relapse | Relapse after CR, CRi, or CRh (excluding cases also meeting the criteria for MLFS):  Any appearance of peripheral blasts, bone marrow blast percentage of ≥5%, or relapsed or new extramedullary leukemia. |
| Unknown | No postbaseline evaluation. |

^a^The outcome was to be captured in the CRF as CR when a patient met the criteria for both CR and CRh.

^b^The outcome was to be captured in the CRF as CRh when a patient met the criteria for both CRi and CRh or both CRh and MLFS.

# **Supplementary Table 2. Summary of Neutrophils and Platelet Counts in the Safety Analysis Set (n=14).**

| Time point | Base line | Cycle 1 Day 2 | Cycle 1 Day 8 | Cycle 1 Day 14 | Cycle 1 Day 22 | Cycle 2 Day 1 | Cycle 2 Day14 | Cycle 3 Day1 | Cycle 4 Day1 | Cycle 5 Day1 | End of Treatment |
| --- | --- | --- | --- | --- | --- | --- | --- | --- | --- | --- | --- |
| Neutrophiles (10^9^/L) | | | | | | | | | | | |
| n | 14 | 13 | 13 | 13 | 12 | 7 | 7 | 5 | 2 | 2 | 12 |
| Mean | 1.27 | 1.89 | 0.65 | 0.43 | 0.54 | 1.64 | 0.39 | 1.56 | 0.79 | 0.05 | 1.96 |
| SD | 3.278 | 3.789 | 1.455 | 0.529 | 0.916 | 2.604 | 0.393 | 2.420 | 0.49 | 0.059 | 5.358 |
| Min | 0.0 | 0.0 | 0.0 | 0.0 | 0.0 | 0.0 | 0.0 | 0.1 | 0.4 | 0.0 | 0.0 |
| Median | 0.13 | 0.30 | 0.16 | 0.22 | 0.15 | 0.21 | 0.39 | 0.81 | 0.79 | 0.05 | 0.16 |
| Max | 12.5 | 13.2 | 5.4 | 1.8 | 3.1 | 6.3 | 1.0 | 5.8 | 1.1 | 0.1 | 18.9 |
| Platelet (10^9^/L) | | | | | | | | | | | |
| n | 14 | 13 | 13 | 13 | 12 | 7 | 7 | 5 | 2 | 2 | 12 |
| Mean | 54.6 | 48.8 | 32.5 | 23.8 | 26.8 | 49.3 | 22.1 | 27.4 | 73.5 | 83.5 | 32.7 |
| SD | 36.72 | 34.06 | 20.32 | 16.04 | 14.52 | 33.08 | 8.32 | 18.19 | 68.59 | 65.76 | 27.92 |
| Min | 9 | 8 | 10 | 7 | 12 | 13 | 9 | 7 | 25 | 37 | 11 |
| Median | 43.0 | 35.0 | 26.0 | 21.0 | 24.0 | 41.0 | 25.0 | 25.0 | 73.5 | 83.5 | 28.0 |
| Max | 115 | 106 | 78 | 70 | 60 | 108 | 30 | 47 | 122 | 130 | 114 |

SD: standard deviation, Min: minimum, Max: maximum

# **Supplementary Table 3. *TP53* status**

| **Dose (mg)** | **Patient #** | **Blood** | | **Bone marrow** | |
| --- | --- | --- | --- | --- | --- |
|  |  | **Pretreatment** | **End of treatment** | **Pretreatment** | **End of treatment** |
| 90 | 01 | Mutant | NA | Mutant | NA |
|  | 02 | Wild | Wild | Wild | NA |
|  | 03 | Wild | Wild | Wild | Mutant |
|  | 04 | Wild | Wild | Wild | Wild |
| 120 | 05 | Wild | Mutant | Mutant | Mutant |
|  | 06 | Wild | NA | Wild | NA |
|  | 07 | Wild | Wild | Wild | NA |
|  | 08 | Wild | Mutant | Wild | Mutant |
|  | 09 | Wild | Wild | Wild | Wild |
|  | 10 | Wild | Wild | Wild | Wild |
| 160 | 11 | Mutant | Mutant | Mutant | Mutant |
|  | 12 | Wild | Wild | Wild | Wild |
|  | 13 | Wild | Wild | Wild | NA |
|  | 14 | Wild | Wild | Wild | NA |

NA: not applicable

# **Supplementary Figure 1. Treatment schedule and study design**

## **A. Doses and dosing schedules**





This dose-escalation study with starting dose of 90 mg once daily (QD) was conducted with 14/28-day was schedule A. Schedules B, C, and D were to be conducted as necessary. The study drug was administered once daily on days 1–14, 1–7, and 1–21 in schedules A, B, and D, followed by a 14-, 21-, and 7-day rest period, respectively, in a 28-day cycle. The study drug was to be administered once daily on days 1–3 in schedule C, followed by an 11-day rest, in a 14-day subcycle, which would be repeated twice in a 28-day cycle.

## **B. Study Design**





# **Supplementary Figure 2. Scatter plots of milademetan dose and AUC for the individual patients**





*AUC_8h_* area under the plasma concentration–time curve during 8 h, *C_max_* maximum plasma concentration

# **Supplementary Figure 3****. Waterfall plot of best percent change from baseline blast count in bone marrow aspirate**





Patients (*n* = 12)

Baseline is defined as the last non-missing value taken before the first dose of the study drug. The best percent change from baseline blast count for each patient is defined as the most decreased blast count in bone marrow aspirate. The TP53 status was defined as the baseline results.
